# The Impact of Mouse Passaging of *Mycobacterium tuberculosis* Strains prior to Virulence Testing in the Mouse and Guinea Pig Aerosol Models

**DOI:** 10.1371/journal.pone.0010289

**Published:** 2010-04-21

**Authors:** Paul J. Converse, Kathleen D. Eisenach, Sue A. Theus, Eric L. Nuermberger, Sandeep Tyagi, Lan H. Ly, Deborah E. Geiman, Haidan Guo, Scott T. Nolan, Nicole C. Akar, Lee G. Klinkenberg, Radhika Gupta, Shichun Lun, Petros C. Karakousis, Gyanu Lamichhane, David N. McMurray, Jacques H. Grosset, William R. Bishai

**Affiliations:** 1 Department of Medicine, Center for Tuberculosis Research, Johns Hopkins University, Baltimore, Maryland, United States of America; 2 Department of Pathology, University of Arkansas for Medical Sciences, Little Rock, Arkansas, United States of America; 3 Department of Microbial and Molecular Pathogenesis, Texas A&M University, College Station, Texas, United States of America; Charité-Universitätsmedizin Berlin, Germany

## Abstract

**Background:**

It has been hypothesized that the virulence of lab-passaged *Mycobacterium tuberculosis* and recombinant *M. tuberculosis* mutants might be reduced due to multiple in vitro passages, and that virulence might be augmented by passage of these strains through mice before quantitative virulence testing in the mouse or guinea pig aerosol models.

**Methodology/Principal Findings:**

By testing three *M. tuberculosis* H37Rv samples, one deletion mutant, and one recent clinical isolate for survival by the quantitative organ CFU counting method in mouse or guinea pig aerosol or intravenous infection models, we could discern no increase in bacterial fitness as a result of passaging of *M. tuberculosis* strains in mice prior to quantitative virulence testing in two animal models. Surface lipid expression as assessed by neutral red staining and thin-layer chromatography for PDIM analysis also failed to identify virulence correlates.

**Conclusions/Significance:**

These results indicate that animal passaging of *M. tuberculosis* strains prior to quantitative virulence testing in mouse or guinea pig models does not enhance or restore potency to strains that may have lost virulence due to in vitro passaging. It is critical to verify virulence of parental strains before genetic manipulations are undertaken and comparisons are made.

## Introduction

Serial in vitro passaging of pathogenic bacteria and viruses is a classical method for obtaining attenuated strains that can be used either as vaccines or as safe laboratory model strains to study basic functions of the pathogen [1]. Examples in tuberculosis include the Bacille Calmette-Guérin (BCG) vaccine strain and H37Ra obtained by intentional serial culture of virulent *Mycobacterium bovis* and the variably virulent *M. tuberculosis* H37 strain, respectively [2], [3].

The question of whether to maintain the virulence of tubercle bacilli through animal passage may have its roots in the personal, legal, and scientific disputes between Robert Koch and his student Emil von Behring in the late 19^th^ and early 20^th^ century [4], [5], [6]. Koch reviewed the “law of increasing virulence,” originally enunciated by Casimir Davaine in studies of anthrax, that an initial animal passage could “select” bacteria that were more pathogenic than those in the original tissue source [7]. Koch does not appear to have pursued this line of enriching bacterial virulence in his studies of tuberculosis. In fact, as an adherent of the monomorphic school of bacteriology [8], [9], he emphasized the stability of the organism from host to culture to new host [10]. Von Behring sought to define a therapy for tuberculosis after Koch's failure to cure patients with tuberculin. Anti-toxin therapy, for which von Behring received the first Nobel Prize ever awarded in Medicine in 1901, was rapidly recognized to have no likelihood of success against TB as it had had against diphtheria. Seeking a vaccine against cattle tuberculosis in order to prevent transmission of bovine bacilli through the digestive tract, which he proposed to nearly always occur in childhood and to be a prerequisite for adult pulmonary disease [6], von Behring reported in his Nobel Lecture [11] that tubercle bacilli derived from human sputum were not “unharmful” for cows but that they lost their virulence “through long-continued culture [6.5 years [2]] in the laboratory.” These bacilli could be restored to “considerable virulence in cattle” after passage through goats on his Marburg farm [5], [11]. Earlier that same year Koch had proposed in London that there may be differences in the bacteria that cause human and bovine tuberculosis [12]. Koch's Nobel lecture in 1905 [4] insisted on the primacy of the pulmonary route of infection from human to human, continued to dismiss the significance of bovine TB for humans, and only reluctantly yielded on this point at the close of Sixth International Conference on Tuberculosis in 1908 in Washington, DC. There, Theobald Smith successfully made the case for the distinct nature of the bovine bacillus, insisted, with Ravenel and Arloing, on its impact on child health, and initiated a campaign for its eradication in cattle in North America [4], [7]. While the research into practical preventive or therapeutic treatments of tuberculosis of both Berhing (immune milk) and Koch (tuberculin) was ultimately unsuccessful, Behring's vaccine work and concepts of attenuation of *M. tuberculosis*, based in part on the work of Pasteur with anthrax and rabies, almost certainly influenced that of Calmette and Guérin – an influence that was never acknowledged, apparently due to bitter experiences arising from World War I [5].

A century later, specifically over the last 20 years, tools to genetically manipulate *M. tuberculosis* have been widely used. Quantitative virulence comparison of genetically manipulated strains together with their parental wild type strains is a widely used technique for the identification of specific genetic determinants of virulence. However, the process of generating such mutants often begins with parent strains that have been repeatedly passaged in vitro and the process itself involves multiple cycles of in vitro growth which may diminish virulence in a non-specific manner by selecting variants that are best adapted to in vitro conditions. In addition, the host strains used for the manipulation may themselves have undergone prolonged in vitro culture. Because of these concerns, some investigators routinely passage recombinant strains through mice or other animals before conducting a definitive quantitative virulence assay in an animal model [13]. Pre-passaging recombinant mycobacteria through mice or other animals is costly and time-consuming and may also result in unwanted attenuating changes. Importantly, the necessity of pre-passage of *M. tuberculosis* through an animal host has not been scientifically validated. Therefore, we conducted a controlled comparison of the virulence of identical strains passaged and non-passaged in both the mouse and guinea pig tuberculosis models.

Our results show no evidence of enhanced virulence of wild-type *M. tuberculosis* strains or a deletion mutant in mice or guinea pigs after mouse passaging or reduced virulence in strains that have not been passaged.

## Materials and Methods

### Mouse passaging

*M. tuberculosis* strains (0.2 ml) at early-to-mid log phase in Middlebrook 7H9 broth supplemented with 10% oleic acid-albumin-dextrose-catalase (OADC) (Difco) and 0.1% Tween 80 were injected into the lateral tail vein of Swiss mice, and lungs were harvested approximately 10 days after infection. Lung homogenates were serially diluted and plated on Middlebrook 7H10 or 7H11 agar supplemented with 10% OADC. After 2–3 weeks, 5–10 colonies were collected and sub-cultured in 7H9 broth for 5–7 days and intravenous infection was repeated. After this second mouse passage the isolate was grown in 7H9 broth to an od_600_ of 1.0, divided into 1 ml aliquots and frozen at −70°C with the addition of 10% glycerol.

### Animal infections

Three *M. tuberculosis* H37Rv samples, one deletion mutant, and one recent clinical isolate were tested in mouse intravenous or mouse or guinea pig aerosol infection models. One H37Rv strain (termed JHU, obtained from the Johns Hopkins Hospital clinical microbiology laboratory [14]) had been passaged multiple times through mouse lungs after intravenous infection. A second H37Rv strain (TAMU) obtained from ATCC (#27294; preparation described in [15]) was cultured once in Middlebrook 7H9 (M7H9) and frozen for up to 2–3 years before use. A viable count (typically, 2×10^7^ cfu/ml) of the frozen/thawed and briefly sonicated suspension was used to calculate the dilution for the desired infectious dose in physiological saline to be put in the Collison 3-jet nebulizer (#MRECN24) and exposure of guinea pigs in a Madison chamber [16]. The third H37Rv strain (the parent for a *dosR* deletion mutant) was obtained from D. Sherman (Seattle, WA) and passaged twice through mouse lungs. We also had the *dosR* deletion mutant in the pre-passage and twice-passaged state. A final strain tested was SA294 (from Drs. Theus and Eisenach), a recent clinical isolate with 15 IS*6110* bands detected by restriction fragment length polymorphism analysis and found in a single case that was unpassaged and twice-passaged in mice. Female mice (6 weeks old either BALB/c, our standard strain or, on request, C57BL/6, Charles River Laboratories, Wilmington, MA) were exposed to aerosol containing *M. tuberculosis* in a Middlebrook inhalation exposure system (Glas-Col, Terre Haute, IN) that resulted in ∼2–3 log_10_ *M. tuberculosis* implanted on day 1. All protocols were approved by the Institutional Animal Care and Use Committees at Johns Hopkins University (# MO04M380 and MO05M168) or at Texas A&M University (#2006-96).

### CFU and histopathology analysis

Mouse lungs were obtained after sacrifice and homogenized. Serial dilutions of the homogenate were plated on selective Middlebrook 7H11 plates (Becton-Dickinson, Sparks, MD) containing antibiotics to prevent the growth of contaminating organisms. Similarly, the lower right lobe of guinea pig lungs was used for cfu determinations on Middlebrook 7H10 plates. The lower left lobe was placed in 10% formalin and shipped to JHU for histopathological analysis of hematoxylin and eosin-stained sections.

### Lung pathology

The number of low power (20×) fields was counted for each specimen. Within each field, the number of granulomas was also tabulated permitting the calculation of the number of granulomas per low-power microscopic field. Because the size and extent of necrosis of each granuloma varies, a subjective determination on a scale of 1–4 of disease severity was also assessed so that both quantitative and qualitative measures could be used to describe the extent of tissue damage in a manner similar to the method used in a recent publication [17]. The identity of the specimen was shielded from the scorer.

### Surface Lipid Profile analysis

Neutral red assays were carried out as described previously [18], [19]. Briefly, colonies from selected strains were collected and placed in an Eppendorf tube containing 1 ml of 50% methanol, washed twice and kept for 1 hr at 37°C before incubation with 0.002% neutral red in barbital buffer. Color of test strains and H37Rv (red) and H37Ra (yellow) controls were compared after incubation and again after standing at room temperature overnight. Assessment of phthiocerol dimycoserosate (PDIM) was carried out using minor adaptations from previous studies (e.g.,[18]). Briefly, strains were grown in broth to an od_600_ of 1.5 and the cell pellet was inactivated by autoclaving. Lipids were extracted with 2∶1 chloroform/methanol. A portion of the lower organic phase was applied onto a thin layer chromotography (TLC) plate and run in a hexane:diethylether: acetic acid solvent system. The TLC plate was treated with 10% phosphomolybdate in ethanol, heated, and the plate was scanned.

## Results

### Unpassaged and twice-passaged clinical isolate shows similar multiplication in BALB/c mouse lungs

**Figure 1:**
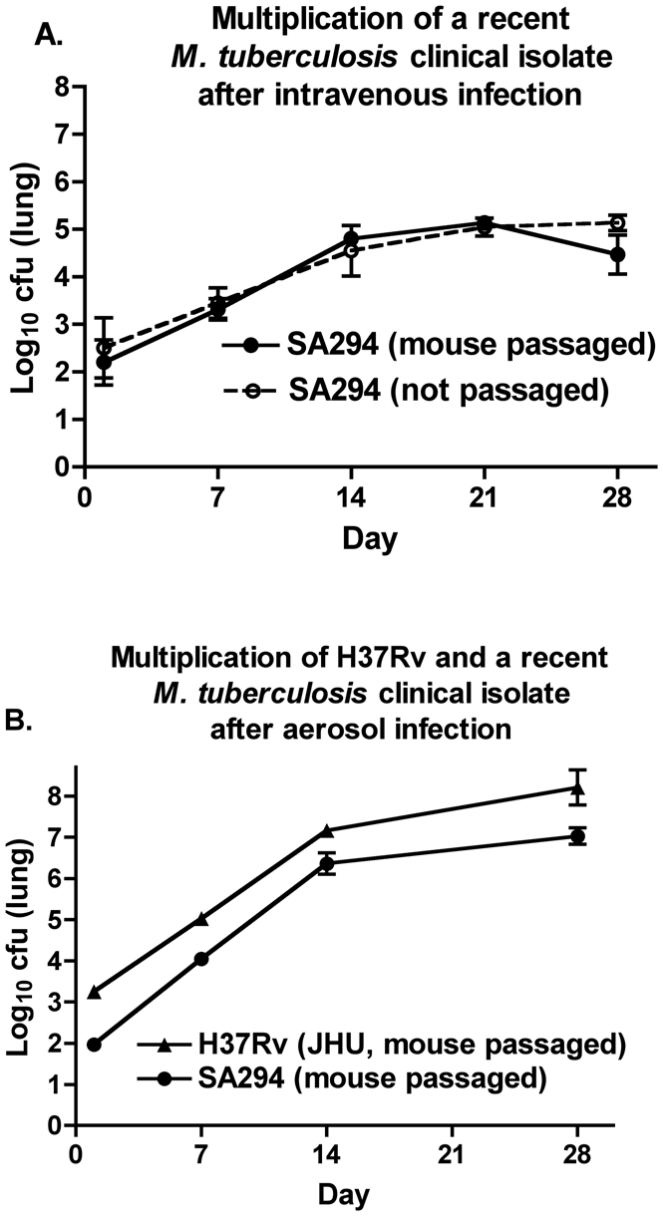
A. Multiplication of mouse-passaged and -unpassaged *M. tuberculosis* clinical isolate in BALB/c mice. A. Growth in lungs of the isolate following intravenous infection. B. Growth in lungs following aerosol infection of mouse-passaged clinical isolate in comparison with regularly mouse-passaged H37Rv strain (JHU) in BALB/c mice. Errors are SD.

In order to evaluate the effect of mouse passaging as described in the Methods section on a recent slow-growing clinical isolate, SA294, we compared the multiplication in lungs of the unpassaged isolate to the mouse passaged strain using 3 BALB/c mice/group in the mouse intravenous infection model. As may be seen in Figure 1A, implantation was similar as assessed on day 1 and both strains multiplied at similar rates until day 28 with cfu levels determined at weekly intervals. We then compared the passaged SA294 strain with the JHU H37Rv strain that has been regularly passaged in mice in the mouse aerosol model with 6 BALB/c mice/group. Although the implantation dose was lower for the recent clinical isolate, the rates of growth in the two strains were parallel (Fig. 1B). These data suggest that this recent clinical isolate was not attenuated in its ability to multiply in mice and that intravenous passage of the strain through mice did not result in an enhanced ability to multiply in BALB/c mouse lungs.

### Unpassaged and twice-passaged H37Rv parent strain and *dosR* deletion mutant show similar multiplication in C57BL/6 mouse lungs

**Figure 2:**
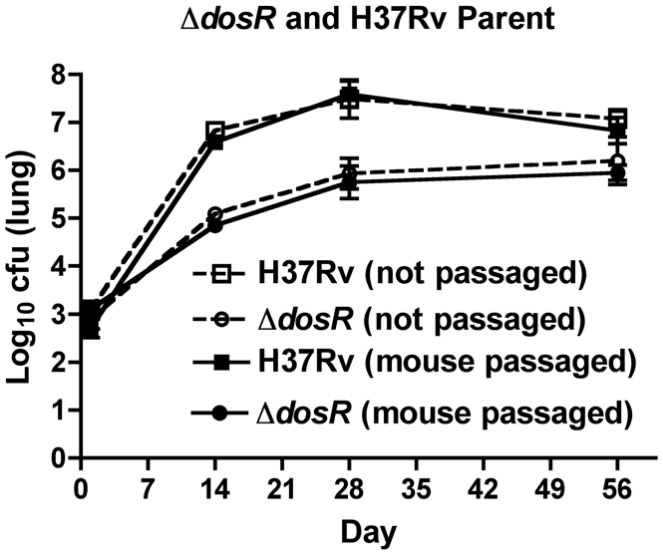
Comparison of bacterial multiplication in the lungs of C57BL/6 mice by passaged and unpassaged *dosR* deletion mutants and their parental *M. tuberculosis* H37Rv strain following aerosol infection. Errors are SD.

Using the mouse aerosol model with 5 C57BL/6 mice/group, we compared multiplication of an H37Rv strain and a *dosR* deletion mutant constructed from it, using isolates that had or had not been mouse passaged. C57BL/6 mice were used to test the hypothesis that Δ*dosR* was hypervirulent and this should be evaluated in a relatively resistant mouse strain [20]. All strains had similar day 1 implantation. The parental strain multiplied considerably better than did the mutant strain, as assessed by lung CFU counts at days 14, 28, and 56, but there were no differences between the passaged and unpassaged strains (Fig. 2). Dissemination to the spleen was also not enhanced in the mouse-passaged strains (data not shown). These data indicate that mouse passaging had no impact on the ability of the wild-type or mutant strain to implant and multiply in mouse lungs or to spread to an extrapulmonary site.

### Guinea pig lung pathology with type strains of H37Rv from different institutions

**Figure 3:**
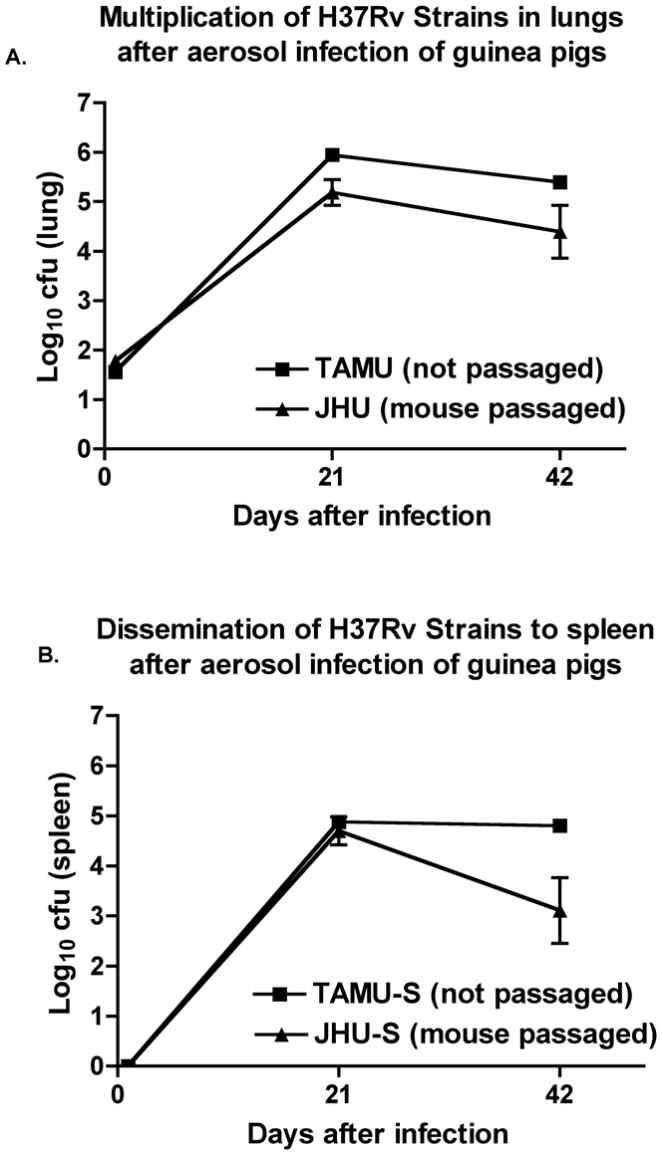
Mouse passaged JHU *M. tuberculosis* H37Rv strain compared to in vitro propagated TAMU H37Rv strain in guinea pigs after aerosol infection. Errors are SEM.

Many institutions have a type strain of *M. tuberculosis* obtained in the remote past, which is used as the standard reference in animal virulence testing in their laboratories. To quantify the virulence of two H37Rv strains used extensively over long periods of time at two such institutions, we conducted comparative testing of the JHU and TAMU H37Rv strains in the guinea pig aerosol model. The JHU H37Rv strain was twice mouse passaged before being divided into aliquots and frozen prior to use, while the TAMU strain after receipt from the ATCC was grown once in large quantity in vitro, aliquoted, and then frozen prior to use. Quantitative organ CFU analysis showed nearly comparable growth in guinea pig lungs (Fig. 3A). By two-way ANOVA analysis, there were no statistically significant differences between the JHU and TAMU strains at day 1 or week 3 while at week 6 the difference in the lungs was of borderline statistical significance (p = 0.044). Spleen CFU counts of TAMU and JHU strains (Fig. 3B) were not different at week 3 but were higher for the TAMU strain at week 6 (p<0.01).

**Figure 4:**
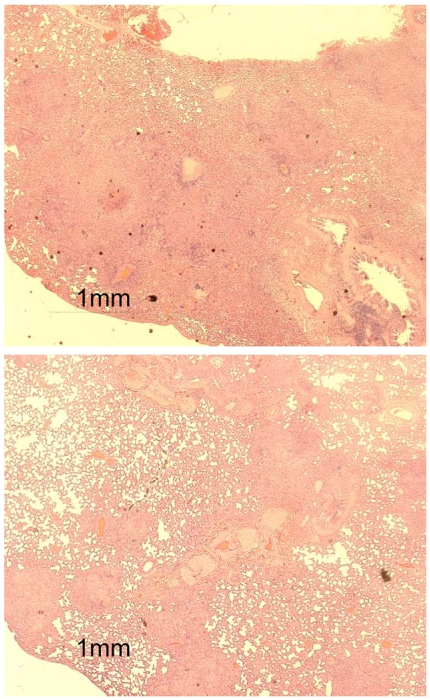
Comparison of inflammatory granulomatous pathology in the lungs of guinea pigs three weeks after aerosol infection with one of two H37Rv strains (unpassaged TAMU, top, and mouse-passaged JHU, bottom).

In addition, we conducted a lung pathology assessment using the scoring system described in the Methods section. Lung pathology was somewhat more severe in guinea pigs infected with the unpassaged TAMU strain (Table 1) based on the number of granulomas per field or the subjective quantitative scoring system. Although both the TAMU and JHU strains induced large numbers of granulomas, the lung granulomas were larger and more often coalesced in TAMU strain-infected guinea pigs and thereby occupied a greater area per low-power microscopic field (Fig. 4). Overall, in the guinea pig aerosol model, the TAMU H37Rv strain, obtained from the ATCC, propagated in Middlebrook broth and frozen, was either comparable or more virulent than the mouse-passaged JHU strain when evaluated in terms of lung CFU counts, spleen CFU counts, or lung pathology.

**Table 1:** Comparative lung histopathology in guinea pigs infected with *M. tuberculosis* H37Rv with different histories of in vitro and in vivo passaging.

H37Rv Strain	Week	Mean # granulomas	granulomas/field	Mean Score (1–4)
TAMU*	3	30	>13	3.25
JHU**	3	26.5	10.6	2.5
TAMU*	6	16	6.4	3.5
JHU**	6	19.5	7.8	2.4

* Not passaged in mice.

** Passaged in mice.

### Lipid profile analysis

All strains were positive for neutral red and all strains expressed PDIMs (data not shown). Lipid phenotyping was therefore not informative in these experiments.

## Discussion

Serial in vitro culture has been used since the time of Pasteur (reviewed in Smith, 1988) to attenuate microbial pathogens for use as vaccines or defined laboratory strains. The H37 clinical isolate was obtained from a 19-year old male patient at the Trudeau sanatorium in New York, in 1905. Steenken and Gardner [3] described the media used at Trudeau during the time, until 1922, in which the isolate maintained its virulence for rabbits and guinea pigs. The critical component appeared to be a peptone imported from Germany. By the time the supply was exhausted, the company was no longer in business and substitution with a domestic peptone resulted in a marked, abrupt, but variable, loss of virulence in the strain. Eventually, avirulent H37Ra and virulent H37Rv were dissociated, using different culture media, from this semi-virulent *M. tuberculosis* H37 in 1934 [3], [21]. However, even at the time that H37Rv was made the type strain at the ATCC, Kubica *et al*. [15] noted that many cultures of H37Rv were no longer virulent for common laboratory animal hosts and recommended a form of the Proskauer-Beck medium most suitable for virulence maintenance, underlining the importance of a slightly alkaline pH of 7.4.

In his Nobel Lecture, Emil von Behring noted that tubercle bacilli directly obtained from humans were not “unharmful” for cattle, that they lost virulence during long-continued culture, but could be restored to full cattle virulence by passage through goats [11]. Calmette, the co-discover of attenuated *M. bovis*, or BCG, wrote in 1920 that virulence testing required the use of strains directly derived from infected organs or after a single culture after passage in guinea pigs [2]. Investigations on variability of virulence of tubercle bacilli were published in the 1950s [22], [23], [24]. Notably, Steenken reported, in a review [25], that animal passaging had never resulted in reversion of an avirulent strain. However, there were some cases in which attenuated bacilli, probably containing a virulent subpopulation, may have preferentially propagated after animal passaging or after culture in synthetic media. Guinea pig testing in the 1950s suggested that strains maintained in vitro were less virulent for guinea pigs (J. Grosset, personal communication). Isoniazid-resistant *M. tuberculosis*, was also reported to be less virulent for guinea pigs [26]. In 1988, Grosset and Ji [13] recommended that a well-characterized, drug-susceptible strain “whose virulence is maintained through regular passages in the mouse, is the strain of choice.” Reports from other contemporary tuberculosis investigators vary in the particulars of the best procedure to use but also support this principle of virulence maintenance through mouse passaging [27], [28]. We have been unable to find studies directly comparing passaged and unpassaged strains. Recently, Ahmad et al. reported results consistent with ours when using the passaged JHU and non-passaged TAMU strains of H37Rv in guinea pigs [29].

Mouse passaging has been proposed as a technique to eliminate less fit phenotypic variant subpopulations that may emerge during prolonged in vitro cultivation [30]. It has been thought that the less virulent variants would fail to survive due to immunological mechanisms present in the mouse. These would necessarily be components of innate immunity due to the short time (<2 weeks) from infection to the harvest of lung tissue. Alternatively, there may be a deficiency in the ability of the less virulent subpopulations to obtain nutrients after injection into the host as proposed by Pierce *et al* [24]. Others have hypothesized that survival of bacteria to the chronic phase in a resistant mouse strain should select for more virulent strains [31]. Finally, it is possible that under the conditions used, in vitro passaging at least for modest periods does not select for less virulent variants that multiply less vigorously or fail to survive after animal infection.

We hypothesized that defined *M. tuberculosis* mutants and their H37Rv parent strains, would have reduced virulence and could be restored to full virulence by passage through mice before testing in the mouse or guinea pig aerosol models. We expected that mouse passaging would eliminate the less fit (i.e., less virulent) subpopulations. As described above, however, the passaging procedures used on the strains compared to date had no impact on the virulence phenotype observed in mice or guinea pigs. In fact, as would have been predicted by the late Frank Collins [21], someone had already “been there, done that.” Arnold Rich [32] cited nearly parallel passaging experiments done at the Trudeau Institute [33] and at Johns Hopkins [34] in guinea pigs over a 10-year period in the 1920s with the R1 strain that was known to have reduced virulence, but not to be avirulent. Essentially none of the isolates after each passage was markedly different from the parental strain in its ability to cause progressive disease. On the other hand, a fully virulent isolate rapidly lost virulence through serial in vitro culture. If the latter isolate was passaged in guinea pigs, from the beginning, it retained full virulence.

One limitation of our study is that our endpoints were limited to organ CFU survival and in some cases tissue pathology. We did not compare the passaged and unpassaged strains for differences in time-to-death. Time-to-death differences between genetically defined strains of *M. tuberculosis* mutants have been reported in the setting of indistinguishable organ CFU burdens [35], [36]. In addition, we tested only a limited number of passaged and non-passaged strain pairs and our results may not be generalizable to all strains maintained in vitro, particularly for extended periods, prior to use in animals. Finally, for the experiments described, we passaged strains by infecting mice intravenously whereas most of the virulence assays were performed in aerosol infection models. We cannot exclude the possibility that the in vivo passaging would have a greater impact on virulence if the strain is passaged by the same route of infection used in the virulence assay. Similarly, we cannot exclude the possibility that the reduced multiplication of the JHU H37Rv strain relative to the TAMU H37Rv strain in the guinea pig was caused by repeated mouse passaging and selection of characteristics favoring growth in mice that are detrimental to growth in a different microenvironment present in the infected guinea pig lung.

Although there is evidence documented in the literature that loss of neutral red staining is associated with loss of virulence [18], [19], [37] and that loss of PDIM expression is also associated with loss of virulence [30], [38], [39], [40], [41], [42], none of the strains used in this study showed altered lipid expression. Although artificial attenuation may occur with loss of PDIMs due to alteration in one or more of the *pps* or *drr* genes or other regulatory elements [30], our experience has also shown in other experiments that PDIM-negative strains can retain full virulence in animals. Our practice is to evaluate lipid profiles and, if negativity is found in the mutant but not the parent strain to consider that attenuation may occur because of loss of the gene of interest and/or the loss of PDIMs.

Based on our findings, we advocate the generation of mutants in parental strains known to be virulent and then to proceed to direct testing of *M. tuberculosis* strains in animal models, without insisting on a prerequisite for animal passaging. Our data support the practice of banking seed lots of reference strains at −80°C and using them for in vivo virulence testing directly instead of requiring one or two rounds of animal passage prior to virulence testing. Once a strain has lost virulence, restoration through animal passaging appears unlikely to succeed.

## References

[1] Smith H (1988). The development of studies on the determinants of bacterial pathogenicity.. J Comp Pathol.

[2] Calmette A (1920). L'infection bacillaire et la TUBERCULOSE chez l'homme et chez les animaux. Processus d'infection et de defense, Étude biologique et expérimentale..

[3] Steenken W, Gardner LU (1946). History of H37 strain of tubercle bacillus.. Am Rev Tuberc.

[4] Daniel TM (2000). Pioneers of Medicine and their impact on tuberculosis..

[5] Linton DS (2005). Emil von Behring: Infectious disease, immunology, serum therapy..

[6] von Behring E (1903). Combatting tuberculosis.Gessamelte Abhandlungen Neue Folge 1915..

[7] Brock TD (1988). Robert Koch: A Life in Medicine and Bacteriology..

[8] Falk IS, Jordan EO, Falk IS (1928). A theory of microbic virulence.. The Newer Knowledge of Bacteriology and Immunology.

[9] Hadley P, Jordan EO, Falk IS (1928). The dissociative aspects of bacterial behavior.. The Newer Knowledge of Bacteriology and Immunology.

[10] Koch R (1882). The aetiology of tuberculosis.. Berliner Klinische Wochenschrift.

[11] von Behring E (1967). Serum Therapy in Therapeutics and Medical Science.. Nobel Lectures, Physiology or Medicine 1901–1921.

[12] Koch R (1901). An address on the fight against tuberculosis in the light of the experience that has been gained in the successful combat of other infectious diseases.. Br Med J.

[13] Grosset J, Ji B, Gangadharam PRJ, Jenkins PA (1988). Experimental Chemotherapy of Mycobacterial Diseases.. Mycobacteria II, Chemotherapy.

[14] Parrish NM, Kuhajda FP, Heine HS, Bishai WR, Dick JD (1999). Antimycobacterial activity of cerulenin and its effects on lipid biosynthesis.. J Antimicrob Chemother.

[15] Kubica GP, Kim TH, Dunbar FP (1972). Designation of strain H37Rv as the neotype of *Mycobacterium tuberculosis*.. Int J Syst Bacteriol.

[16] Wiegeshaus EH, McMurray DN, Grover AA, Harding GE, Smith DW (1970). Host-parasite relationships in experimental airborne tuberculosis. 3. Relevance of microbial enumeration to acquired resistance in guinea pigs.. Am Rev Respir Dis.

[17] Palanisamy GS, Smith EE, Shanley CA, Ordway DJ, Orme IM (2008). Disseminated disease severity as a measure of virulence of *Mycobacterium tuberculosis* in the guinea pig model.. Tuberculosis.

[18] Andreu N, Gibert I (2008). Cell population heterogeneity in *Mycobacterium tuberculosis* H37Rv.. Tuberculosis (Edinb).

[19] Cardona PJ, Soto CY, Martin C, Giquel B, Agusti G (2006). Neutral-red reaction is related to virulence and cell wall methyl-branched lipids in *Mycobacterium tuberculosis*.. Microbes Infect.

[20] Converse PJ, Karakousis PC, Klinkenberg LG, Kesavan AK, Ly LH (2009). Role of the *dosR-dosS* Two-Component Regulatory System in *Mycobacterium tuberculosis* Virulence in Three Animal Models.. Infect Immun.

[21] Collins FM (1998). Tuberculosis research in a cold climate.. Tubercle and Lung Disease.

[22] Bloch H (1950). Studies on the virulence of tubercle bacilli; the relationship of the physiological state of the organisms to their pathogenicity.. J Exp Med.

[23] Bloch H, Segal W (1956). Biochemical differentiation of *Mycobacterium tuberculosis* grown in vivo and in vitro.. J Bacteriol.

[24] Pierce CH, Dubos RJ, Schaefer WB (1953). Multiplication and survival of tubercle bacilli in the organs of mice.. J Exp Med.

[25] Steenken W (1950). Dissociation of the tubercle bacillus; a review.. Am Rev Tuberc.

[26] Mitchison DA (1954). Tubercle bacilli resistant to isoniazid; virulence and response to treatment with isoniazid in guinea-pigs.. Br Med J.

[27] Kramnik I, Dietrich WF, Demant P, Bloom BR (2000). Genetic control of resistance to experimental infection with virulent *Mycobacterium tuberculosis*.. Proceedings of the National Academy of Sciences.

[28] Scanga CA, Mohan VP, Yu K, Joseph H, Tanaka K (2000). Depletion of CD4(+) T cells causes reactivation of murine persistent tuberculosis despite continued expression of interferon gamma and nitric oxide synthase 2.. J Exp Med.

[29] Ahmad Z, Klinkenberg LG, Pinn ML, Fraig MM, Peloquin CA (2009). Biphasic kill curve of isoniazid reveals the presence of drug-tolerant, not drug-resistant, *Mycobacterium tuberculosis* in the guinea pig.. The Journal of Infectious Diseases.

[30] Domenech P, Reed MB (2009). Rapid and Spontaneous Loss of Phthiocerol Dimycocerosate (PDIM) from *Mycobacterium tuberculosis* Grown in vitro: Implications for Virulence Studies.. Microbiology.

[31] Kramnik I (2007). personal communication..

[32] Rich AR (1951). The Pathogenesis of Tuberculosis..

[33] Cummings DE (1932). The virulence of the attenuated strain of tubercle bacillus R1 after serial passage through previously tuberculin-negative guinea pigs.. Am Rev Tuberc.

[34] Willis HS (1933). The effect of animal passage on the virulence of tubercle bacilli.. Am Rev Tuberc.

[35] Kaushal D, Schroeder BG, Tyagi S, Yoshimatsu T, Scott C (2002). Reduced immunopathology and mortality despite tissue persistence in a *Mycobacterium tuberculosis* mutant lacking alternative sigma factor, SigH.. Proc Natl Acad Sci U S A.

[36] Sun R, Converse PJ, Ko C, Tyagi S, Morrison NE (2004). *Mycobacterium tuberculosis* ECF sigma factor *sigC* is required for lethality in mice and for the conditional expression of a defined gene set.. Mol Microbiol.

[37] Dubos RJ, Middlebrook G (1948). Cytochemical reaction of virulent tubercle bacilli.. Am Rev Tuberc.

[38] Camacho LR, Constant P, Raynaud C, Laneelle MA, Triccas JA (2001). Analysis of the phthiocerol dimycocerosate locus of *Mycobacterium tuberculosis*. Evidence that this lipid is involved in the cell wall permeability barrier.. J Biol Chem.

[39] Camacho LR, Ensergueix D, Perez E, Gicquel B, Guilhot C (1999). Identification of a virulence gene cluster of *Mycobacterium tuberculosis* by signature-tagged transposon mutagenesis.. Mol Microbiol.

[40] Cox JS, Chen B, McNeil M, Jacobs WR (1999). Complex lipid determines tissue-specific replication of *Mycobacterium tuberculosis* in mice.. Nature.

[41] Domenech P, Reed MB, Barry CE (2005). Contribution of the *Mycobacterium tuberculosis* MmpL protein family to virulence and drug resistance.. Infect Immun.

[42] Goren MB, Brokl O, Schaefer WB (1974). Lipids of putative relevance to virulence in *Mycobacterium tuberculosis*: correlation of virulence with elaboration of sulfatides and strongly acidic lipids.. Infect Immun.

